# The impact of multicentric datasets for the automated tumor delineation in primary prostate cancer using convolutional neural networks on ^18^F-PSMA-1007 PET

**DOI:** 10.1186/s13014-024-02491-w

**Published:** 2024-08-07

**Authors:** Julius C. Holzschuh, Michael Mix, Martin T. Freitag, Tobias Hölscher, Anja Braune, Jörg Kotzerke, Alexis Vrachimis, Paul Doolan, Harun Ilhan, Ioana M. Marinescu, Simon K. B. Spohn, Tobias Fechter, Dejan Kuhn, Christian Gratzke, Radu Grosu, Anca-Ligia Grosu, C. Zamboglou

**Affiliations:** 1 Department of Radiation Oncology, Faculty of Medicine, Medical Center – University of Freiburg, University of Freiburg, German Cancer Consortium (DKTK), Partner Site DKTK, Freiburg, Germany; 2 Division of Radiology, German Cancer Research Center (DKFZ), Heidelberg, Germany; 3 Department of Nuclear Medicine, Faculty of Medicine, Medical Center – University of Freiburg, Freiburg, Germany; 4 Department of Radiotherapy and Radiation Oncology, Faculty of Medicine, University Hospital Carl Gustav Carus, TUD Dresden University of Technology, Dresden, Germany; 5 Department of Nuclear Medicine, Faculty of Medicine, University Hospital Carl Gustav Carus, TUD Dresden University of Technology, Dresden, Germany; 6 Department of Nuclear Medicine, German Oncology Center, European University Cyprus, Limassol, Cyprus; 7 Department of Medical Physics, German Oncology Center, European University Cyprus, Limassol, Cyprus; 8 Department of Nuclear Medicine, University Hospital - Ludwig-Maximilians-Universität, Munich, Germany; 9 Division of Medical Physics, Department of Radiation Oncology, Faculty of Medicine, Medical Center–University of Freiburg, German Cancer Consortium (DKTK), Partner Site DKTK, Freiburg, Germany; 10 Department of Urology, Medical Center–University of Freiburg, Freiburg, Germany; 11 Cyber-Physical Systems Division, Institute of Computer Engineering and Faculty of Informatics, Technical University of Vienna, Vienna, Austria; 12 Department of Computer Science, State University of New York at Stony Brook, Stony Brook, NY USA; 13 Department of Radiation Oncology, German Oncology Center, European University Cyprus, Limassol, Cyprus

## Abstract

**Purpose:**

Convolutional Neural Networks (CNNs) have emerged as transformative tools in the field of radiation oncology, significantly advancing the precision of contouring practices. However, the adaptability of these algorithms across diverse scanners, institutions, and imaging protocols remains a considerable obstacle. This study aims to investigate the effects of incorporating institution-specific datasets into the training regimen of CNNs to assess their generalization ability in real-world clinical environments. Focusing on a data-centric analysis, the influence of varying multi- and single center training approaches on algorithm performance is conducted.

**Methods:**

nnU-Net is trained using a dataset comprising 161 ^18^F-PSMA-1007 PET images collected from four distinct institutions (Freiburg: n = 96, Munich: n = 19, Cyprus: n = 32, Dresden: n = 14). The dataset is partitioned such that data from each center are systematically excluded from training and used solely for testing to assess the model's generalizability and adaptability to data from unfamiliar sources. Performance is compared through a 5-Fold Cross-Validation, providing a detailed comparison between models trained on datasets from single centers to those trained on aggregated multi-center datasets. Dice Similarity Score, Hausdorff distance and volumetric analysis are used as primary evaluation metrics.

**Results:**

The mixed training approach yielded a median DSC of 0.76 (IQR: 0.64–0.84) in a five-fold cross-validation, showing no significant differences (p = 0.18) compared to models trained with data exclusion from each center, which performed with a median DSC of 0.74 (IQR: 0.56–0.86). Significant performance improvements regarding multi-center training were observed for the Dresden cohort (multi-center median DSC 0.71, IQR: 0.58–0.80 vs. single-center 0.68, IQR: 0.50–0.80, p < 0.001) and Cyprus cohort (multi-center 0.74, IQR: 0.62–0.83 vs. single-center 0.72, IQR: 0.54–0.82, p < 0.01). While Munich and Freiburg also showed performance improvements with multi-center training, results showed no statistical significance (Munich: multi-center DSC 0.74, IQR: 0.60–0.80 vs. single-center 0.72, IQR: 0.59–0.82, p > 0.05; Freiburg: multi-center 0.78, IQR: 0.53–0.87 vs. single-center 0.71, IQR: 0.53–0.83, p = 0.23).

**Conclusion:**

CNNs trained for auto contouring intraprostatic GTV in ^18^F-PSMA-1007 PET on a diverse dataset from multiple centers mostly generalize well to unseen data from other centers. Training on a multicentric dataset can improve performance compared to training exclusively with a single-center dataset regarding intraprostatic ^18^F-PSMA-1007 PET GTV segmentation. The segmentation performance of the same CNN can vary depending on the dataset employed for training and testing.

**Supplementary Information:**

The online version contains supplementary material available at 10.1186/s13014-024-02491-w.

## Introduction

Primary prostate cancer (PCa) represents one of the most common cancer types in men with a prevalence of up to 15% in industrialized nations [1]. In the treatment of localized prostate cancer, radiotherapy holds a pivotal position. As there has been an upsurge in more patient-centric approaches such as the use of focal radiation dose escalation, a meticulous delineation of the intraprostatic tumor burden is essential [2].

^18^F-PSMA-1007 PET imaging has emerged as a powerful tool for characterizing intraprostatic tumor lesions [3], exhibiting, in some aspects even higher sensitivities compared to prostate multiparametric magnetic resonance imaging (mpMRI) [4].

While manual segmentation can be used for delineating the intraprostatic Gross Tumor Volume (GTV) [5], modern deep learning algorithms, especially convolutional neural networks (CNNs), are significantly transforming the contouring process in radiation oncology across various dimensions [6–10].

In light of this critical shift towards automated, deep learning-based contouring, the feasibility of CNN-based autocontouring has been demonstrated for both ^68^ Ga-PSMA-11 and ^18^F-PSMA-1007 PET in prior work [11–13].

However, these studies primarily focused on the practical aspects of intraprostatic GTV segmentation, neglecting crucial technical and data-centric considerations necessary for the successful integration of CNNs into clinical workflows.

Undoubtedly, external validation remains a crucial aspect in the evaluation of deep learning models [14, 15], as the heterogeneity of imaging data across different clinical centers poses a significant barrier to the universal adoption and effectiveness of these technologies. The consideration of multicentric training datasets emerges as an important factor in addressing these challenges, aiming to enhance the robustness and accuracy of CNN-based autocontouring solutions. Therefore, the motivation behind our study is twofold: to critically analyze the performance implications of employing a multicentric approach in the training of deep learning models for intraprostatic GTV delineation while also investigating the potential benefits of integrating institution-specific nuances into the training process. By doing so, we strive to contribute valuable insights into the optimization of deep learning applications in the domain of radiation oncology, ultimately facilitating the advancement of patient-centric treatment strategies that are both effective and adaptable to varied clinical settings.

In this study, we systematically assess the importance of using a multicentric training dataset, evaluating the generalizability and performance of CNNs for intraprostatic GTV delineation on ^18^F-PSMA-1007 PET across different clinical settings inside the nnUNet Framework [16].

Initially, models are trained on datasets from all centers except one and subsequently tested on a cohort from the excluded center. In a second phase, the models are trained using data from a single center and tested on datasets from all other centers. Comparative analysis of the outcomes is performed against results obtained from a dataset that includes mixed data from all centers.

Through the comparison of different training strategies and validation datasets, our research aims to delineate the critical factors that contribute to the success of CNN-based auto contouring in a clinical context, ultimately guiding future developments in the field of radiation oncology.

## Methods

### Patients

A total of 161 patients diagnosed with primary prostate cancer (PCa) from four different medical centers (Freiburg: n = 96, Munich: n = 19, Cyprus: n = 32, Dresden: n = 14) were included in this study. Inclusion criteria were biopsy-proven diagnosis of primary PCa without having received prior treatment at the time of imaging. Local ethics committees from all participating centers granted approval or exemption for the study. Patients with high and intermediate-risk PCa were included retrospectively. The detailed characteristics are shown in Table 1 and have been described in prior work [11]. Imaging characteristics can be found in the supplementary.

**Table 1:** Patient characteristics

Dataset (n = 161)	Freiburg	Cyprus	Munich	Dresden
n	96	32	19	14
Mean age [years] (standard deviation)	69.3 (8.1)	69.2 (7.5)	67.2 (10.9)	70.4 (8)
Median iPSA [ng/ml] (min–max)	14.6 (4.2–164)	10.2 (2.75–167)	10.4 (4.6–465)	16.5 (5–139)
ISUP				
1	5 (5%)	8(25%)	2 (10%)	1 (7%)
2	24 (25%)	4(12.5%)	6 (32%)	2 (14%)
3	29 (30%)	8 (25%)	3 (16%)	4 (29%)
4	21 (22%)	9 (28%)	6 (32%)	2 (14%)
5	17 (18%)	3 (9%)	2 (10%)	3 (21%)
Unknown	–	–	–	2 (14%)
cT stage				
T1	–	11 (34%)	–	4 (29%)
T2	48 (50%)	10 (31%)	12 (63%)	4 (29%)
T3	46 (48%)	9 (28%)	6 (32%)	4 (29%)
T4	2 (2%)	–	1 (5%)	–
Unknown	–	2 (6%)	–	2 (14%)

### Preprocessing

For preprocessing, body weight-adapted standardized uptake value (SUV) PET scans were resampled to a voxel size of 2 × 2 × 2 mm^3^ using B-spline interpolation. The focus was narrowed to the prostate area by cropping to a size of 64 × 64 × 64 voxels based on the prostate contour, enhancing computational efficiency. Input SUV-PET images were further normalized by applying value clipping at a SUV of 20, followed by regular nnU-Net preprocessing [16]. Voxels outside of the prostate were not changed.

### Architecture and training

The nnUNet architecture [16] was utilized, taking SUV PET and prostate contour as input while outputting intraprostatic GTV contours. Training the network involved a specialized approach to accommodate the sparsely labeled dataset. Emphasis was placed on intraprostatic lesions using a weighted cross-entropy (CE) loss, thereby refining the model's focus and accuracy for intraprostatic GTV delineation based on the contour of the prostate. Besides training for 200 epochs, nnUNet default schedule was used, leveraging its established efficacy in medical image segmentation without the need for further hyperparameter optimization.

### Data splitting

**Fig. 1:**
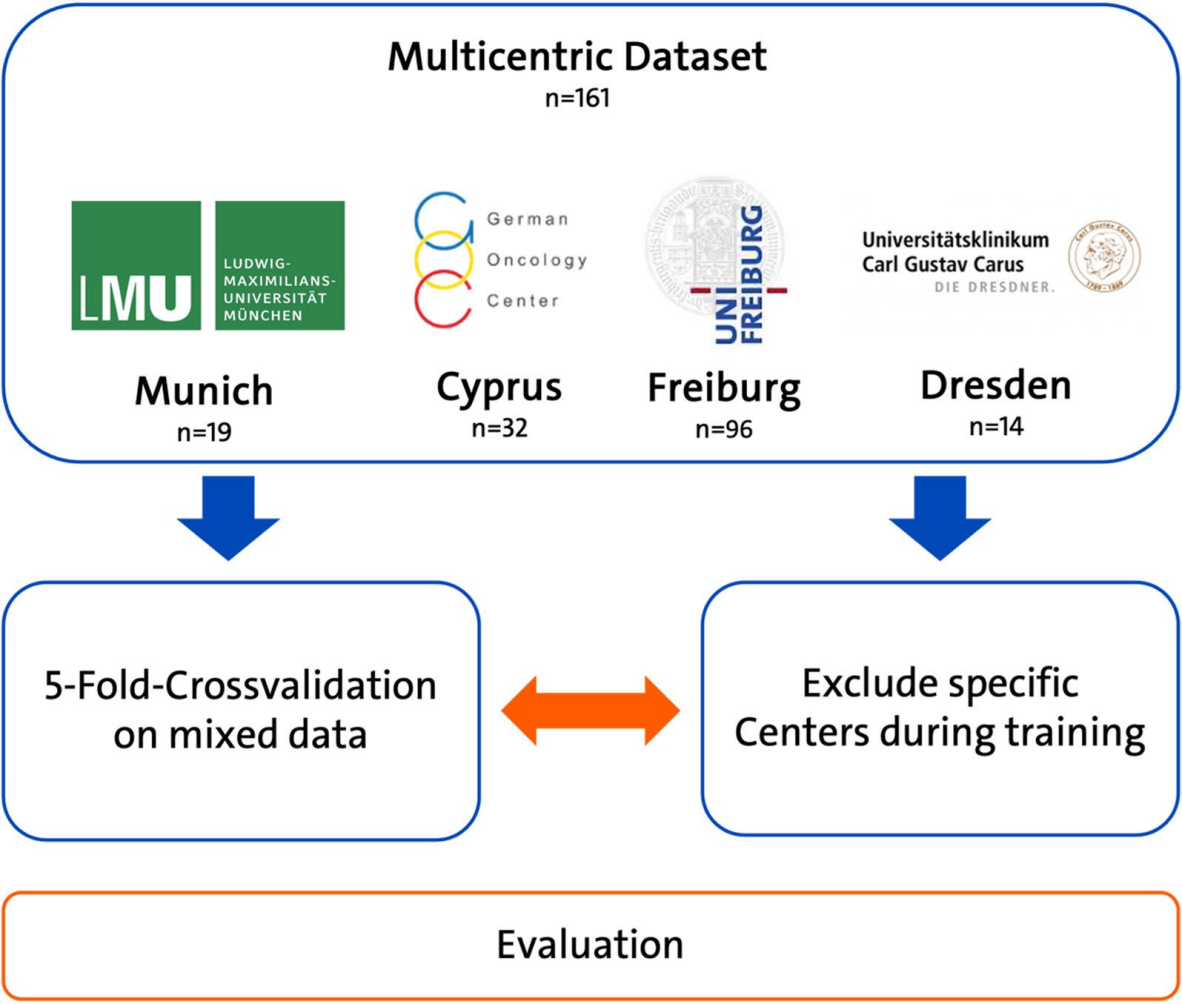
Experimental setup is illustrated, comparing a 5-Fold Cross-Validation with the systematic exclusion of different centers while training. Experiments are conducted within the nnU-Net Framework using Dice Score Similarity Index (DSC), 95% Hausdorff Metric (HD95) and Volumetric analysis for comparison

To compare different types of training data we used five-fold cross-validation as depicted in Fig. 1. For single center training comparison to mixed data, we first performed a split excluding the specific center. To ensure a fair comparison, we further partitioned the split into five subsets that are collectively exhaustive and mutually exclusive of the respective split and added training data from the other centers to reach the same amount of training data. We compared the respective results for the leave-one-center-out approach to an adjusted five-fold cross-validation on the whole dataset for the same respective validation set.

### Evaluation

Evaluation metrics include volumetric Dice Similarity Coefficient (DSC) [17] and Hausdorff Distance 95% (HD95) [18, 19], providing a comprehensive assessment of segmentation accuracy and following metric selection guidelines [18]. Statistical analysis was conducted using the Wilcoxon signed-rank test and a paired t-test with Bonferroni correction, based on the scipy 1.10.1 and statannot 0.6.0 library.

### Contouring

The contouring methodology built upon previously established techniques and the exact process has been described in previous works [5, 11]. A consensus contour was derived from the expertise of two board-certified radiation oncologists, both experienced in intraprostatic GTV contouring and PET image analysis.

## Results

**Fig. 2:**
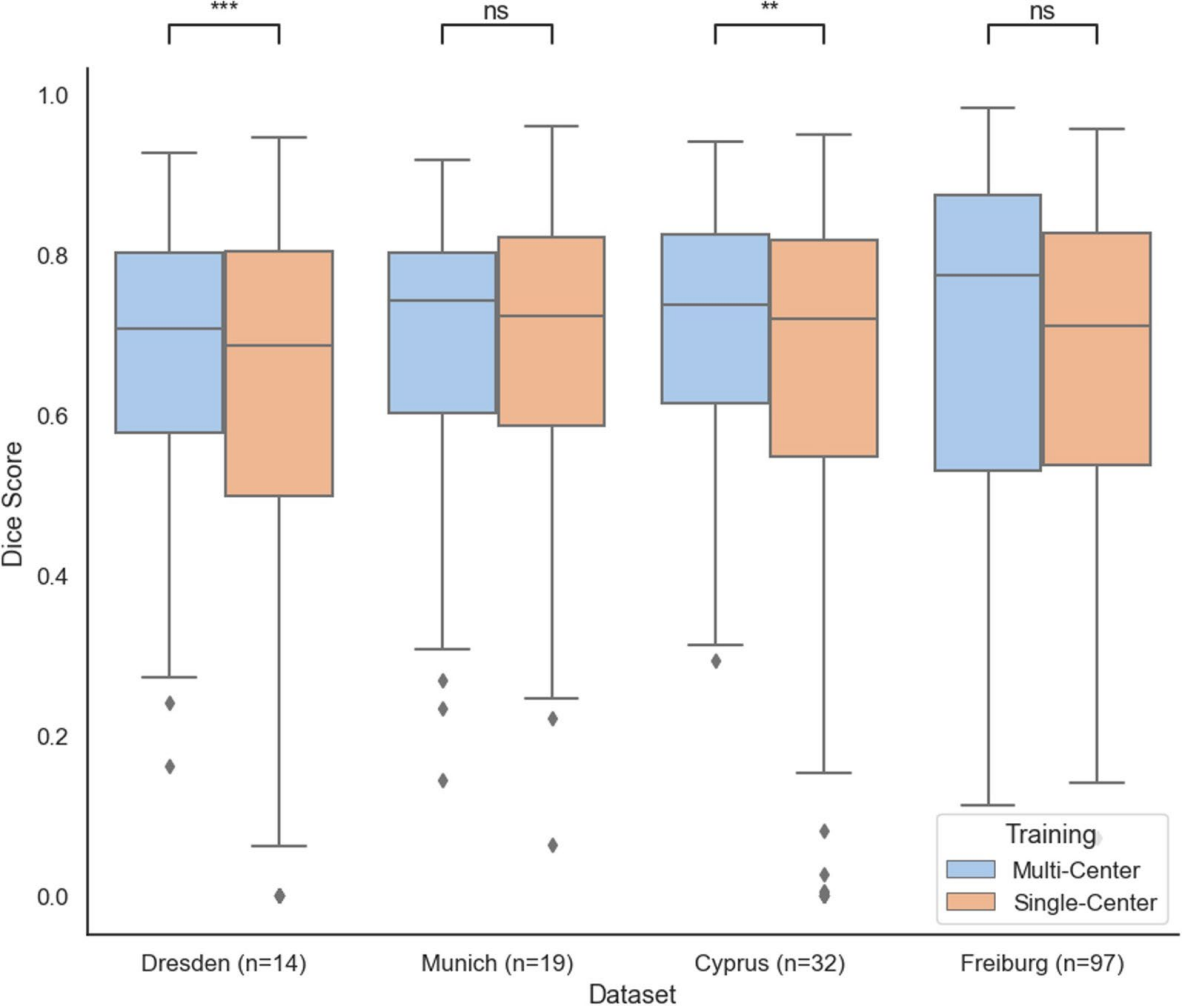
Comparative analysis of training performance: multi-centric vs. single-center showing a significant performance increase for multi-centric training. The figure presents boxplots illustrating the performance outcomes of models trained on a multi-centric dataset (in blue) through cross-validation, contrasted against those trained on a single-center dataset (in orange). Statistical significance is denoted as: ns (not significant, p > 0.05), * (p < 0.01), ** (p < 0.001), and *** (p < 0.0001), indicating a substantial performance enhancement with multi-centric training. Dice score is shown on the y-axis and the respective cohort used for single-center training is shown on the x-axis with the respective sample size denoted as n that was used during multi-center training. Results are reported on the same validation samples for each pair

Comparing single-center to multicentric training, adjusted for sample size, significant performance improvements regarding multi-center training were observed for the Dresden cohort (multi-center median DSC 0.71, IQR: 0.58–0.8 vs. single-center 0.68, IQR: 0.50–0.8, p < 0.001) and Cyprus cohort (multi-center 0.74, IQR: 0.62–0.83 vs. single-center 0.72, IQR: 0.54–0.82, p < 0.01). While Munich and Freiburg cohorts also indicated enhanced performance for multi-center training over single-center training, results showed no statistical significance (Munich: multi-center DSC 0.74, IQR: 0.60–0.80 vs. single-center 0.72, IQR: 0.59–0.82, p > 0.05; Freiburg: multi-center 0.78, IQR: 0.53–0.87 vs. single-center 0.71, IQR: 0.53–0.83, p > 0.05). A comparative analysis is depicted in Fig. 2.

Regarding the 95% Hausdorff Distance (HD95) metric, comparisons between multi-center and single-center training revealed no significant difference across each comparison (p > 0.05). Specifically, the median HD95 values for Center Dresden were 1.94 (IQR: 1.35–3.22) for multi-center training and 1.73 (IQR: 1.00–3.70) for single-center training. For Center Munich, the values were 1.71 (IQR: 1.20–2.90) for multi-center training versus 1.73 (IQR: 1.01–4.10) for single-center training. Center Cyprus reported HD95 values of 1.60 (IQR: 1.14–2.71) for multi-center training compared to 1.41 (IQR: 1.0–4.15) for single-center training. Lastly, Center Freiburg exhibited median HD95 values of 1.41 (IQR: 1.41–2.95) for multi-center training and 1.85 (IQR: 1.14–3.83) for single-center training.

**Fig. 3:**
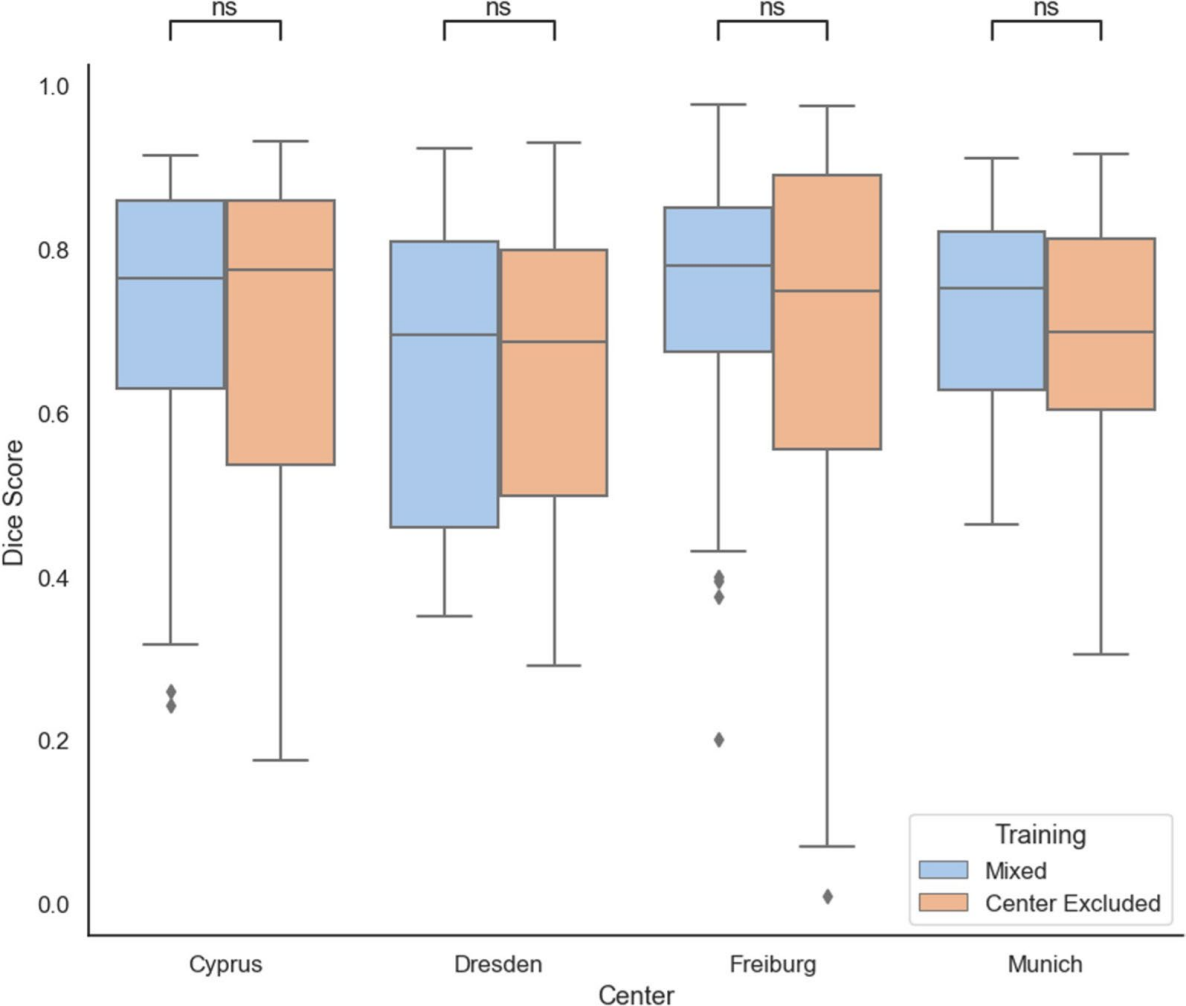
Comparative analysis of training performance: leave-one-center-out vs. mixed training. The figure displays boxplots representing the performance outcomes of models trained using a Leave-One-Center-Out approach (orange) compared to those trained with a mixed cross-validation training strategy (blue). While statistical analysis indicates no significant differences (p > 0.05), slightly higher performance can be noted in most cases for the mixed training approaches. Dice score is shown on the y-axis and the respective cohort used for evaluation is shown on the x-axis. Results are reported on the same validation samples for each pair

Utilizing a five-fold cross-validation on the entire cohort of all centers yielded a median DSC of 0.76 (IQR: 0.64–0.84). When CNNs were trained excluding data from each specific center, results indicated a median DSC of 0.74 (IQR: 0.56–0.86). Statistical analysis revealed no significant difference in DSC values between these two methodologies (p = 0.18). A detailed center-wise analysis is depicted in Fig. 3.

Concerning HD95 metric, cross-validation exhibited a median HD95 of 1.73 mm (IQR: 1–2). Models trained while excluding one center at a time demonstrated a median HD95 of 1.41 mm (IQR: 1–4) on the whole dataset, with these differences reaching statistical significance (p < 0.01).

Volumetric analysis showed a median of 0.87 ml (IQR: 0.41–1.63) for manual contouring, a median of 1 ml (IQR: 0.58–1.60) for cross-validation and a median of 0.86 ml (IQR: 0.43–1.61) for CNNs trained on utilizing a leave-one-center-out approach respectively, showing no statistically significant differences (p > 0.05).

Model training required approximately 40–60 s per epoch. Inference for a single prediction on resampled data was achieved in under one second.

## Discussion

In the present study, we undertook a comprehensive analysis evaluating the ability of CNNs to generalize to novel datasets in the context of intraprostatic GTV delineation for ^18^F-PSMA-1007 PET imaging. Our findings indicate that training the model with datasets from multiple centers significantly improves performance compared to training solely on data from a single center, when compared on an equivalent amount of training data. This improvement is anticipated, as exposure to multicentric data during training enables the model to encounter a broader variety of data representations, thereby enhancing its generalization capacity. Additionally, although the difference in performance between the Leave-One-Center-Out approach and mixed training did not reach the required significance level, the mixed training methodology also exhibited slightly superior performance. Overall, results suggest that integrating data from all centers in the training process, despite sometimes only yielding a small benefit, can contribute to a more robust model by providing a more diverse training dataset.

The difficulty of training deep learning models that perform well on novel, unseen data represents a fundamental challenge within the current landscape of machine learning [23]. Factors such as AI bias [21], shortcut learning [21], distribution shifts [22] and heterogenous acquisition and data annotation [19] further complicate this issue. Even when performing the same method on similar tasks, results can vary greatly depending on the dataset used.

This phenomenon is evident across various studies regarding automated tumor segmentation in PSMA-PET. For intraprostatic GTV segmentation, Kostyszyn et al. (2021) [12] demonstrated that a CNN achieved median DSC of 0.81 to 0.84 across internal and external independent validation cohorts on ^68^ Ga-PSMA-11 and ^18^F-PSMA-1007 PET scans. Adding to these findings, Ghezzo et al. (2023) [13], conducted an independent external validation of Kostyszyn et al.'s method, observing lower median DSC values ranging from 0.72 to 0.77, with mean DSC values between 0.69 and 0.71 for ^68^ Ga-PSMA-11 PET.

Holzschuh et al. (2023) [11], reported a range of median DSC values, from 0.70 to 0.82, for ^18^F-PSMA-1007, ^18^F-DCFPyL, and ^68^ Ga-PSMA-11. Notably, in this study an external validation was conducted independently by another institute.

Leung et al. reported a mean DSC of 0.7 for ^18^F-DCFPyL PET [23], though it is unknown if only intraprostatic or whole-body lesions are considered.

Regarding whole body PSMA PET, Kendrick et al. (2022) [24] reported a median DSC of 0.5 in a single-center study. Huang et al. (2023) [25] report mean DSC values ranging from 0.59 to 0.63 on ^68^ Ga-PSMA-11 PET. Jafari et al. [26] presented results for whole-body ^68^ Ga-PSMA-11 PET, showing voxel-level mean DSC values of 0.65 to 0.7 for different independent centers.

Notably, our results are consistent with previously observed data ranges for automated tumor segmentation in PSMA-PET imaging. Our analysis also reveals that the performance of CNNs in delineating GTV is influenced by the dataset employed for training and testing. This variance also underscores the complexity of machine learning models in adapting to new, unseen data, highlighting the critical importance of well annotated, diverse and representative training datasets to improve model generalization, which is particularly relevant in the context of medical imaging.

However, despite the potential for slight performance decrements, our study also demonstrates that models can achieve commendable performance in certain cases when trained with mixed data from multiple centers, even if the quantity of data is small (n = 19), yielding a median DSC of 0.74. This underscores the data-dependent nature of AI experiments, which can lead to over- or under-estimation of the trained segmentation model's final performance. While the nnU-Net employed in our study aims to maintain hyperparameter invariance to data due to its end-to-end design, this aspect gains particular significance in the context of comparing models that have undergone individual hyperparameter optimization.

Regarding limitations, our study's conclusions are inherently confined to the delineation of intraprostatic GTV using the ^18^F-PSMA-1007 PET tracer within the nnUNet framework. Although, to the best of our knowledge, this represents one of the most extensive cohorts to date concerning ^18^F-PSMA-1007 PET imaging, it is imperative to incorporate more data in future research as cohorts from individual centers are relatively small, necessitating verification of these results across larger cohorts. Moreover, the inherent challenges associated with image segmentation metrics must be acknowledged [18, 27]. Also, tumor size can represent a potential source of bias that could affect segmentation results. In our study, the cohorts comprised patients at different tumor stages, which were not homogenous across different centers. For instance, no stage I patients were included in the Freiburg and Munich cohorts while present in other cohorts. This resulted in potential variability in tumor sizes across the groups.

Additionally, the exploration of alternative deep learning architectures is warranted in subsequent studies, given that our analysis was limited to the nnU-Net architecture. While our cohorts offer a diverse clinical spectrum, results may also vary across different patient collectives.

Overall, our research highlights the importance of multicentric training datasets in enhancing the generalization capabilities of CNNs, underscoring the relationship between dataset diversity and the performance of machine learning models.

## Conclusion

CNNs trained for auto contouring intraprostatic GTV in ^18^F-PSMA-1007 PET on a diverse dataset from multiple centers mostly generalize well to unseen data from other centers. Training with a multicentric dataset can improve performance compared to training exclusively on single-center datasets regarding intraprostatic ^18^F-PSMA-1007 PET GTV segmentation. The segmentation performance of the same CNN can vary depending on the dataset employed for training and testing.

### Supplementary Information

Additional file 1

## Data Availability

No datasets were generated or analysed during the current study.

## Abbreviations

AI: Artificial intelligence
CNN: Convolutional neural network
CE: Cross-entropy
CT: Computer tomography
DSC: Dice similarity coefficient
GTV: Gross tumor volume
HD95: Hausdorff distance 95%
IQR: Interquartile range
mpMRI: Multiparametric magnetic resonance imaging
PCa: Primary prostate cancer
PET: Positron emission tomography
SUV: Standardized uptake value
