# Supplementary material for: The impact of multicentric datasets for the automated tumor delineation in primary prostate cancer using convolutional neural networks on 18F-PSMA-1007 PET
Source: Radiat Oncol. 2024 Aug 7;19:106. doi: 10.1186/s13014-024-02491-w (PMC11304577; doi:10.1186/s13014-024-02491-w)
Supplement: Supplementary file 1 — Additional file 1 [file 13014_2024_2491_MOESM1_ESM.docx]

**Supplementary**

| Supplementary Table 1:  Training and Testing splits for Leave-One-Center-Out Approach |
| --- |
| \| Training \| \| Testing \| \|  \| Analysis \| \| --- \| --- \| --- \| --- \| --- \| --- \| \| Center \| n \| Center \| n \|  \| n \| \| Dresden, Munich, Freiburg \| 129 \| Cyprus Cohort \| 32 \|  \| 32 \| \| All centers excluding Cyprus subset 1 \| 129 \| Cyprus subset 1 \| 6 \| Combine subsets (union) resulting in whole cohort \| 32 \| \| All centers excluding Cyprus subset 2 \| 129 \| Cyprus subset 2 \| 6 \| \| All centers excluding Cyprus subset 3 \| 129 \| Cyprus subset 3 \| 6 \| \| All centers excluding Cyprus subset 4 \| 129 \| Cyprus subset 4 \| 7 \| \| All centers excluding Cyprus subset 5 \| 129 \| Cyprus subset 5 \| 7 \| \| Dresden, Munich, Cyprus \| 65 \| Freiburg Cohort \| 96 \|  \| 96 \| \| All centers excluding Freiburg subset 1 \| 65 \| Freiburg subset 1 \| 19 \| Combine subsets (union) resulting in whole cohort \| 96 \| \| All centers excluding Freiburg subset 2 \| 65 \| Freiburg subset 2 \| 19 \| \| All centers excluding Freiburg subset 3 \| 65 \| Freiburg subset 3 \| 19 \| \| All centers excluding Freiburg subset 4 \| 65 \| Freiburg subset 4 \| 19 \| \| All centers excluding Freiburg subset 5 \| 65 \| Freiburg subset 5 \| 20 \| \| Cyprus, Munich, Freiburg \| 147 \| Dresden Cohort \| 14 \|  \| 14 \| \| All centers excluding Freiburg subset 1 \| 147 \| Dresden subset 1 \| 2 \| Combine subsets (union) resulting in whole cohort \| 14 \| \| All centers excluding Freiburg subset 2 \| 147 \| Dresden subset 2 \| 3 \| \| All centers excluding Freiburg subset 3 \| 147 \| Dresden subset 3 \| 3 \| \| All centers excluding Freiburg subset 4 \| 147 \| Dresden subset 4 \| 3 \| \| All centers excluding Freiburg subset 5 \| 147 \| Dresden subset 5 \| 3 \| \| Cyprus, Dresden, Freiburg \| 142 \| Munich Cohort \| 19 \|  \| 19 \| \| All centers excluding Munich subset 1 \| 142 \| Munich subset 1 \| 3 \| Combine subsets (union) resulting in whole cohort \| 19 \| \| All centers excluding Munich subset 2 \| 142 \| Munich subset 2 \| 4 \| \| All centers excluding Munich subset 3 \| 142 \| Munich subset 3 \| 4 \| \| All centers excluding Munich subset 4 \| 142 \| Munich subset 4 \| 4 \| \| All centers excluding Munich subset 5 \| 142 \| Munich subset 5 \| 4 \| |
| The table shows the data partitioning for each training split within the single-Leave-One-Center-Out approach. Mutually exclusive subsets were used if possible. Samples designated for testing were not utilized in the training of the corresponding split. Analyses and comparisons were consistently performed on the same samples. |
|  |

| Supplementary Table 2:  Training and Testing splits for Single Center Training Approach |
| --- |
| \| Training \| \| Testing \| \|  \|  \| \| --- \| --- \| --- \| --- \| --- \| --- \| \| Center \| N \| Center \| N \|  \| N Analysis \| \| Dresden \| 14 \| Cyprus Munich Freiburg \| 147 \|  \| 147 \| \| Dresden subset 1 + other centers subset 1 \| 14 \| Cyprus Munich Freiburg excluding other centers subset 1 \| 29 \| Combine subsets (union) resulting in whole cohort \| 147 \| \| Dresden subset 2 + other centers subset 2 \| 14 \| Cyprus Munich Freiburg excluding other centers subset 2 \| 29 \| \| Dresden subset 3 + other centers subset 3 \| 14 \| Cyprus Munich Freiburg excluding other centers subset 3 \| 29 \| \| Dresden subset 4 + other centers subset 4 \| 14 \| Cyprus Munich Freiburg excluding other centers subset 4 \| 30 \| \| Dresden subset 5 + other centers subset 5 \| 14 \| Cyprus Munich Freiburg excluding other centers subset 5 \| 30 \| \| Freiburg \| 96 \| Freiburg Cohort \| 65 \|  \| 65 \| \| Freiburg subset 1 rand + other centers subset 1 \| 96 \| Cyprus Munich Dresden excluding other centers subset 1 \| 13 \| Combine subsets (union) resulting in whole cohort \| 65 \| \| Freiburg subset 2 rand + other centers subset 2 \| 96 \| Cyprus Munich Dresden excluding other centers subset 2 \| 13 \| \| Freiburg subset 3 rand + other centers subset 3 \| 96 \| Cyprus Munich Dresden excluding other centers subset 3 \| 13 \| \| Freiburg subset 4 rand + other centers subset 4 \| 96 \| Cyprus Munich Dresden excluding other centers subset 4 \| 13 \| \| Freiburg subset 5 rand + other centers subset 5 \| 96 \| Cyprus Munich Dresden excluding other centers subset 5 \| 13 \| \| Cyprus \| 32 \| Dresden Munich Freiburg \| 129 \|  \| 129 \| \| Cyprus subset 1 + other centers subset 1 \| 32 \| Dresden Munich Freiburg excluding other centers subset 1 \| 25 \| Combine subsets (union) resulting in whole cohort \| 129 \| \| Cyprus subset 2 + other centers subset 2 \| 32 \| Dresden Munich Freiburg excluding other centers subset 2 \| 26 \| \| Cyprus subset 3 + other centers subset 3 \| 32 \| Dresden Munich Freiburg excluding other centers subset 3 \| 26 \| \| Cyprus subset 4 + other centers subset 4 \| 32 \| Dresden Munich Freiburg excluding other centers subset 4 \| 26 \| \| Cyprus subset 5 + other centers subset 5 \| 32 \| Dresden Munich Freiburg excluding other centers subset 5 \| 26 \| \| Munich \| 19 \| Dresden Cyprus Freiburg \| 142 \|  \| 142 \| \| Munich subset 1 + other centers subset 1 \| 32 \| Dresden Cyprus Freiburg excluding other centers subset 1 \| 28 \| Combine subsets (union) resulting in whole cohort \| 142 \| \| Munich subset 2 + other centers subset 2 \| 32 \| Dresden Cyprus Freiburg excluding other centers subset 2 \| 28 \| \| Munich subset 3 + other centers subset 3 \| 32 \| Dresden Cyprus Freiburg excluding other centers subset 3 \| 28 \| \| Munich subset 4 + other centers subset 4 \| 32 \| Dresden Cyprus Freiburg excluding other centers subset 4 \| 29 \| \| Munich subset 5 + other centers subset 5 \| 32 \| Dresden Cyprus Freiburg excluding other centers subset 5 \| 29 \| |
| The table delineates the data partitioning for each training split within the single-center approach. Where the single-center cohort contained excess samples, random sampling was necessary while otherwise, mutually exclusive subsets were used. Samples designated for testing were not utilized in the training of the corresponding split. Analyses and comparisons were consistently performed on the same samples. |

| Supplementary Table 3: Scanner Characteristics |
| --- |
| \| **Center** \| **Freiburg** \| **Munich** \| **Dresden** \| **Cyprus** \| \| --- \| --- \| --- \| --- \| --- \| \| PET imaging system (Type, Manufacturer) \| 64-slice Vereos PET/CT and Gemini TF Big Bore (Philips Healthcare, USA) \| GE Discovery 690 and Siemens (Biograph mCT and Biograph 64) \| Biograph Vision 600, Siemens Healthcare GmbH \| Discovery IQ2 PET/CT system (4 rings; 16 slices) of General Electric \| \| Tracer \| ^18^F-PSMA-1007 \| ^18^F-PSMA-1007 \| ^18^F-PSMA-1007 \| ^18^F-PSMA-1007 \| \| Post injection time \| 2 hours \| 1 hour \| ca. 90 min \| 2 hours \| \| CT \| 120 kV, 100–400 mAs (dose modulation) \| 120 kV, 200–240 mAs \| 12 kV, 11 mAs \| 120 kV, 15–220 mAs (dose modulation) \| \| Contrast enhancement \| mixed \| + \| - \| - \| \|  \|  \|  \|  \|  \| |
| The table shows the respective imaging characteristics for the scanners used in this study [15]. |
|  |
